# Anatomical variants of the acromioclavicular joint influence its visibility in the standard MRI protocol in patients aged 18–31 years

**DOI:** 10.1007/s00276-022-02973-0

**Published:** 2022-07-06

**Authors:** Fredrik Helleberg, Piotr Sobecki, Rafał Józwiak, Paweł Szaro

**Affiliations:** 1 Department of Radiology, Institute of Clinical Sciences, Sahlgrenska Academy, University of Gothenburg, Gothenburg, Sweden; 2 Department of Musculoskeletal Radiology, Sahlgrenska University Hospital, Göteborgsvägen 31, 431 80 Gothenburg, Sweden; 3 Applied Artificial Intelligence Laboratory, National Information Processing Institute, Warsaw, Poland; 4 Faculty of Mathematics and Information Science, Warsaw University of Technology, Warsaw, Poland; 5 Department of Descriptive and Clinical Anatomy, Medical University of Warsaw, Warsaw, Poland

**Keywords:** Acromioclavicular joint, Magnetic resonance imaging, Anatomy, Acromion, Shoulder pain

## Abstract

**Purpose:**

Visualization of a structure in orthogonal planes is essential for correct radiological assessment. The aim was to assess the utility of the standard MRI protocol for the shoulder in the assessment of the acromioclavicular joint (ACJ).

**Methods:**

A total of 204 MRI scans of the shoulder were re-reviewed. Visibility of the ACJ in orthogonal planes was assessed, and the type of acromion and the angle between the ACJ and the glenoid cavity were assessed by two observers.

**Results:**

Agreement in the assessment of ACJ visibility was moderate to substantial. The ACJ was visible in the three anatomical views in 48% (confidence interval [CI] 95% = [41–54%]) of the examinations, and no significant difference regarding gender or age was noticed. The mean angle between the ACJ and the glenoid cavity was 41.12 deg. CI95% = (39.72, 42.53) in the axial plane, 33.39 deg. CI95% = (31.33, 35.45) in the coronal plane and 52.49 deg. CI95% = (50.10, 54.86) in the sagittal plane. When the ACJ was visible in the sagittal and axial planes, significant differences were noticed in the remaining planes (*p* < .05).

**Conclusion:**

Anatomical variations of the ACJ influence its visibility in the standard MRI protocol for examining the shoulder, making this protocol insufficient for ACJ assessment in the examined population.

## Introduction

The acromioclavicular joint (ACJ) is formed between the acromial end of the clavicle and the acromion of the scapula. It is part of the shoulder girdle and plays an important role in allowing motion of the arm [14]. The articular space of the incongruous articular surfaces contains an intraarticular disk [3, 9]. When viewed in the coronal section, the ACJ is normally angled so that it reaches from superolateral to inferomedial [7]. The joint orientation in the remaining anatomical planes and its influence on the radiological diagnostic of the ACJ is not described in the available literature.

The acromion, ACJ, the coracoacromial ligament form the superior border of the subacromial space. Overuse or osteoarthritis of the ACJ can cause osteophytes on the inferior surface of the ACJ, which, in turn, affects the rotator cuff [18]. Bone marrow edema, inferior joint distension and impression on the supraspinatus muscle are predictive MRI signs in patients with symptomatic ACJ osteoarthritis [26]. Subacromial impingement syndrome is the most common cause of shoulder pain seen in general practice and in the younger population [25]. Choice of treatment for impingement syndrome depends on the underlying pathology, so detailed diagnostic imaging is sometimes crucial. Choo et al. demonstrated the difficulty of discriminating between symptomatic and asymptomatic ACJ using standard shoulder MRI [2]. MRI examination of the ACJ is also used to assess injuries to ligaments around the ACJ, which enables a more detailed classification of ACJ dislocations [1].

Today, there is no established MRI protocol available that is specific for the examination of the ACJ. Instead, the same MRI protocol that is used to visualize the shoulder joint is applied. The coronal section is put parallel to the supraspinatus tendon. The sagittal section is then put perpendicular to the coronal. The transverse cut is put without any angle from the cranial part of the caput of the humerus and downwards. The protocol is developed to visualize the tendons of the rotator cuff. The difficulty of visualizing the ACJ with a routine MRI scan has been recognized by other authors, and there have been approaches to develop a protocol adapted for examination of the ACJ [5, 23].

Pathology in the ACJ occurs even in the young population and, if left untreated at an older age, may result in damage to the rotator cuff. The current study aims to systematically evaluate how often the ACJ is seen in routine MRI, and to the best of our knowledge, similar research has not been conducted before. Degenerative changes can affect ACJ morphology. It is known from previous studies that the ACJ space becomes narrower with increasing age [22]. Given the early onset of degenerative changes in the ACJ, we chose to focus on examinations performed on younger patients to eliminate the effect of degenerative changes in and around the ACJ. In addition, it is known that in the examined age group, pain in the shoulder area more often originates from the ACJ, while rotator cuff tears observed in older patients may follow degenerative changes [19].

The primary aim of the study is to investigate in what extension it is possible to evaluate the ACJ based on a standard MRI of the shoulder in patients aged 18–31 years. Furthermore, the aim is to establish whether there are any differences in how well the joint is imaged between different groups based on gender, age and joint morphology.

## Material and methods

### Inclusion and exclusion criteria

Two hundred and four shoulder MRI examinations of patients aged between 18 and 31 were included and re-reviewed. The number allows calculating the frequency of a visible joint cavity with a confidence interval (CI) of ± 7%, which is regarded as a reasonable consideration. MRI examinations were randomly chosen from examinations performed in the Västra Götaland region between 2015 and 2020. All examinations were clinically indicated. Inclusion criteria are presented in Table 1. The presence of fracture (12 cases), infection (2 cases), tumor (4 cases), poor quality of MRI (16 cases) or osteosynthesis material (16 cases) in the examined area excluded the scan (Table 1). The MRI protocol may somewhat vary between cases because imaging was performed on different MRI 1.5 T machines. The most frequently used protocol is shown in Table 2.

**Table 1:** Inclusion and exclusion criteria for MRI examinations

Inclusion criteria	Exclusion criteria
MRI of the shoulder that included the ACJ	Osteosynthesis material in the examined area
Patients aged between 18 and 31 years	Signs of infection in the examined area
	Presence of tumor in the examined area
	Fracture in the examined area
	Poor quality of MRI

*ACJ *acromioclavicular joint, *MRI *magnetic resonance imaging

**Table 2:** Most used sequences in the re-reviewed examinations

Sequence	TE	TR (range)	FOV	Voxel size	Time
PD TSE SPAIR ax	30 ms	2700–5000 ms	140 × 140 x 04 mm	0.50 × 0.50 mm	03:41
T1 TSE Cor	9 ms	450–750 ms	160 × 140 x 69 mm	0.45 × 0.58 mm	00:45
T2 TSE SPAIR Sag	70 ms	3000–5000 ms	140 × 140 x 84	0.50 × 0.50 mm	03:30
T2 TSE SPAIR Cor	45 ms	3000–5000 ms	140 × 140 x 87 mm	0.52 × 0.52 mm	03:54

*Ax *axial section, *Cor *coronal section, *FOV *field of view, *PD *proton density, *SPAIR *spectral adiabatic inversion recovery, *Sag *sagittal, *TE *echo time, *TR *repetition time, *TSE *turbo spin echo. Slice thickness 3 mm

### Radiological assessment

Two observers reviewed the MRI examinations (PS and FH). The final decision was made by consensus. The inclusion of MRI examinations in the study was supervised by a musculoskeletal radiologist (PS) with 7-year experience in musculoskeletal radiology. MRI examinations were reviewed using a dedicated radiological station with AGFA© PACS (Picture Archiving and Communication System).

### Data collection

The age and gender of every patient were noted. Visibility of the joint cavity was assessed in the axial, coronal and sagittal planes. The joint cavity was considered visible if the space between the subchondral bone and cartilage of the acromion and the clavicle was seen.

**Fig. 1:**
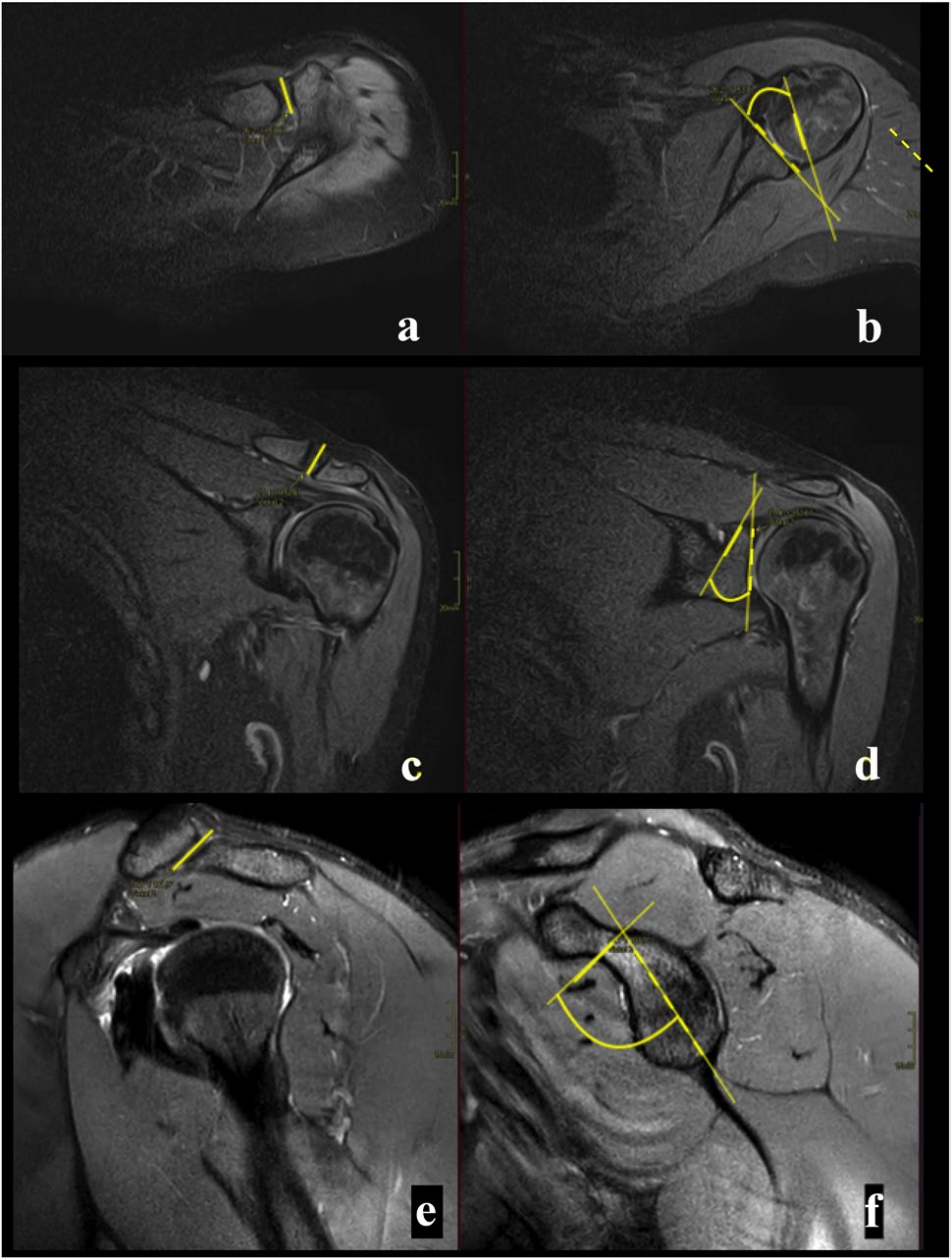
The angles between the acromioclavicular joint (yellow) and the glenoid (dashed line) was measured in axial (**a** and **b**), coronal (**c** and **d**) and sagittal section (**e** and **f**). The joint space is visible on all presented sections (**a**–**f**). A and **b**—proton density (PD)-weighted turbo spin echo (TSE) Spectral Attenuated Inversion Recovery (SPAIR), **b**–**f** —T2-weighted TSE SPAIR

The angle between the ACJ and the glenoid was measured in the three anatomical planes. The glenoid cavity was used as a reference plane in relation to the orientation of the ACJ. In the axial plane, the angle was calculated between the ACJ and the anterior–posterior axis of the central part of the glenoid cavity (Fig. 1a–b). The coronal angle was measured between the ACJ cavity and the superior–inferior axis of the central part of the glenoid seen in the coronal section (Fig. 1c–d).

The angle in the sagittal plane was determined by measuring between the joint cavity axis and the cranial–caudal axis of passing via the central part of the glenoid cavity seen in the sagittal section (Fig. 1e–f).

When the joint cavity was not visible in a certain plane, the angle was not measured. The type of acromion was evaluated according to Bigliani [16]. The analysis of acromion type was performed according to the classic criteria described in previous studies [15, 16]. Cohen's kappa was used to calculate the degree of agreement in the type of acromion between two observers, and the final decision was made by consensus.

### Groups in the study

We divided our group into two subgroups to see whether there were any significant differences between them. The first group included patients aged 18–24 years, while the other subgroup was aged 25–31.

### MRI sequences

Since the examinations were performed in different clinics and during a six-year period, there was variation in the sequences used. The most common sequences are shown in Table 2. Slice thickness was 3 mm without interslice gaps. A dedicated shoulder coil was used for MRI acquisition. The patient was in the supine position, and a dedicated coil with additional elastic wedge-shaped cushions suited to the shape of the shoulder was used to secure the standard position.

### Statistical analysis

The D'Agostino–Pearson Omnibus Test was used to test whether a sample differed from a normal distribution. The Fisher test was used to test for association between two categorical variables. The *T*-test was used to determine whether there was a significant difference between the means of variables that followed the normal distribution, while the Mann–Whitney *U* test was used to compare the medians of variables that did not follow the normal distribution. For variables that did not follow the normal distribution, bootstrapping was used to estimate CIs. Cohen's kappa was used to calculate the degree of agreement in the type of acromion between two observers. Interpretation of Cohen's kappa results was made according to Landis and Koch [13]. ICC was used to evaluate agreement in angle assessment between observers. Interpretation of ICC was made according to Koo et al. [12]. The statistical significance threshold was set to 0.05. The data were analyzed using SPSS Statistics software version 22.0.

### Ethics

The Swedish Ethical Review Authority approved the study and waived the need for informed consent (Dnr 2020–05,954). The study was conducted in compliance with the Declaration of Helsinki. The anonymization of patient data ensured data protection following the European General Data Protection Regulation. The data were recorded in a password-protected secure database.

## Results

There were 145 males and 59 females in the included group. The mean age was 24.09 years (range 18–31, standard deviation 3.72). The number of patients aged 18–24 years was *n* = 101, while the number of patients aged 25–31 was *n* = 103. Age was not of normal distribution. The right shoulder was examined in 115 cases, and the left shoulder was examined in 89 cases.

**Fig. 2:**
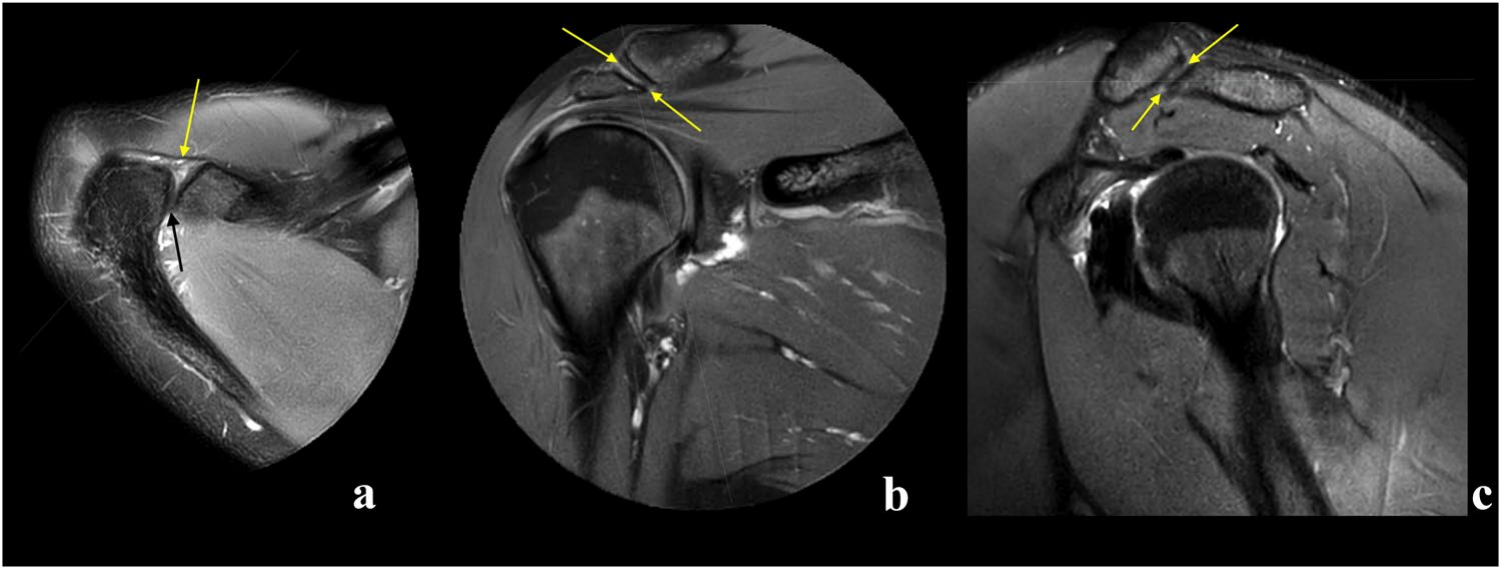
Examples of the visible joint cavity in axial (**a**), coronal (**b**) and sagittal view **c**, respectively (arrows). Three different patients, **a** a 23-year-old patient with the shoulder pain, **b** an 18-year-old patient with suspicion of the subacromial impingement, **c** a 28-year-old patient with suspicion of the supraspinatus tear. **A**—proton density (PD)-weighted turbo spin echo (TSE) Spectral Attenuated Inversion Recovery (SPAIR), **b** and **c**—T2-weighted TSE SPAIR

**Fig. 3:**
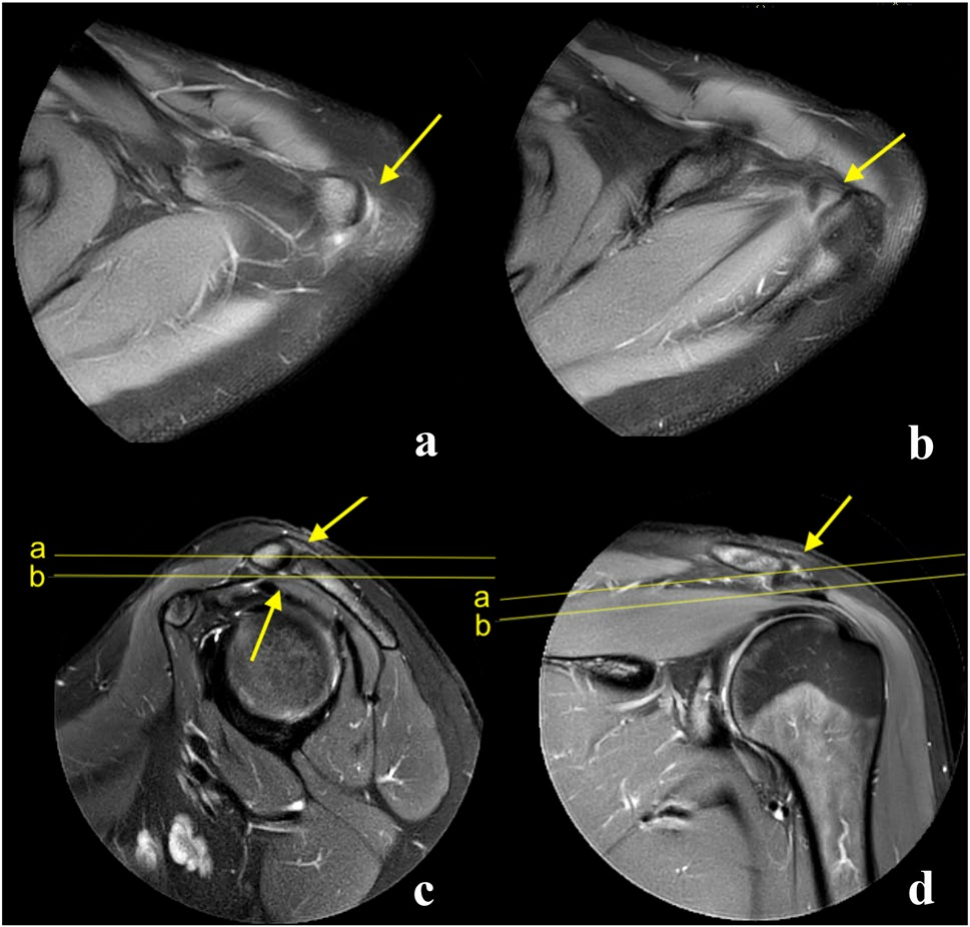
Example of an examination where the joint cavity is not visible in axial plane. The joint space is not visible on the axial plane (**a** and **b**) while visible on the sagittal plane (**c**) and not visible on the coronal plane (**d**). The localization of the joint is marked by arrows. An 18-year-old patient with suspicion of the subacromial bursitis. **A** and **b**—proton density (PD)-weighted turbo spin echo (TSE) Spectral Attenuated Inversion Recovery (SPAIR), **c** and **d**—T2-weighted TSE SPAIR

**Fig. 4:**
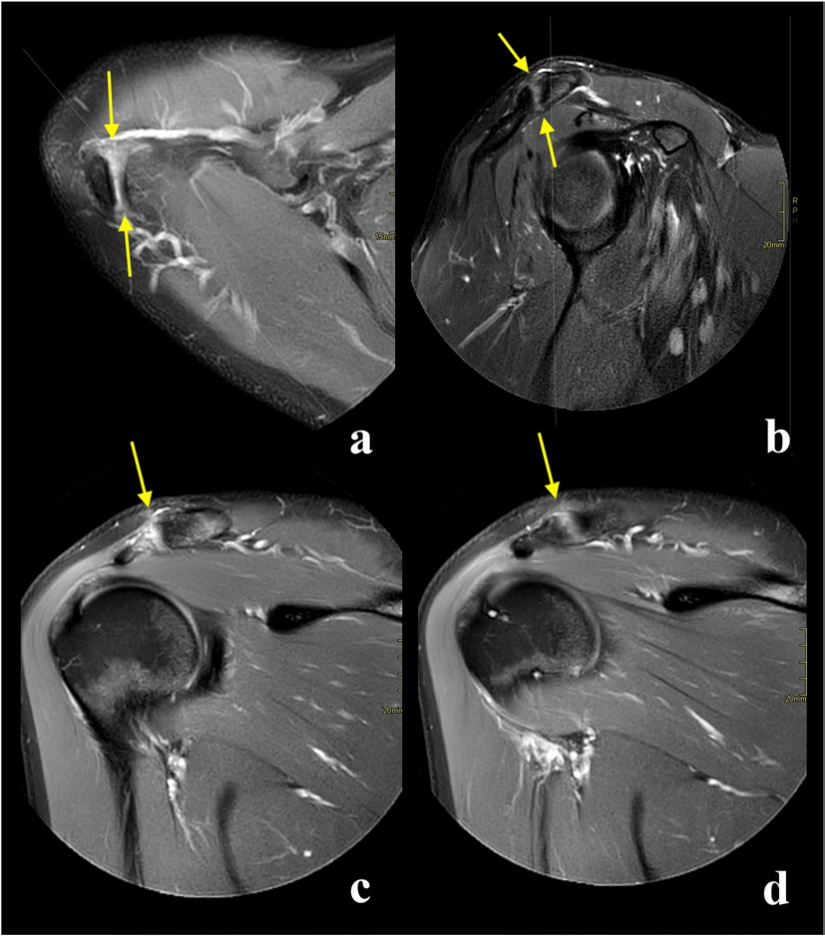
Example of an examination where the joint cavity is not visible in coronal plane. The arrows show the localization of the acromioclavicular joint. The joint space is visible on the axial plane (**a**) and sagittal plane (**b**). The joint space is not visible on the coronal plane (**c** and **d**), localization of the joint is marked by arrows. A 26-year-old patient with suspicion of supraspinatus tear. **A**—proton density (PD)-weighted turbo spin echo (TSE) Spectral Attenuated Inversion Recovery (SPAIR), **b**, **c** and **d**—T2-weighted TSE SPAIR

**Fig. 5:**
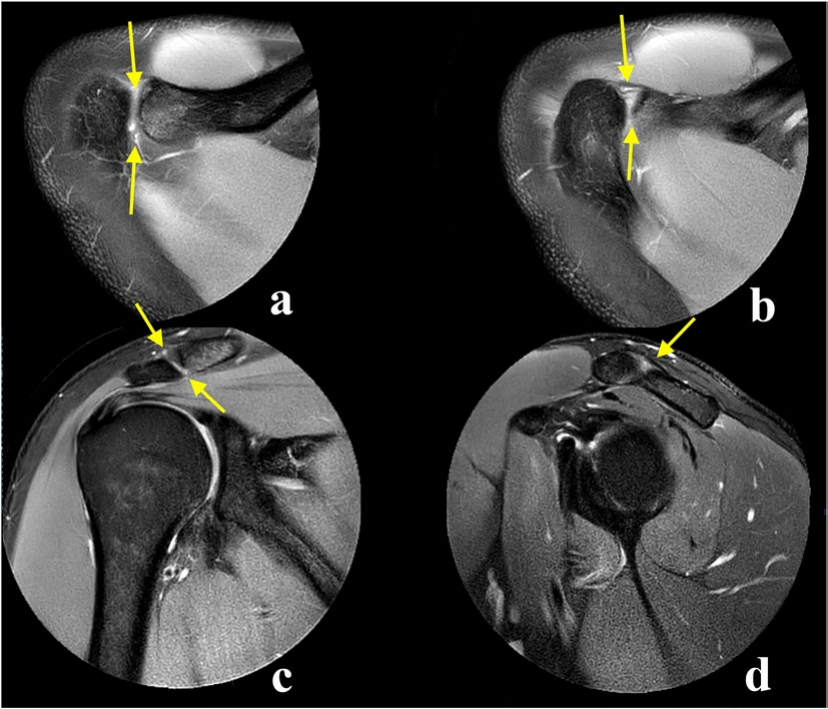
Example of an examination where the joint cavity is not visible in sagittal view. The arrows show the localization of the acromioclavicular joint. The joint space is visible on the axial plane (**a** and **b**) and coronal plane (**c**). The joint space is not visible on the sagittal plane (**d**), localization of the joint is marked by arrows. A 30-year-old patient with suspicion of subacromial bursitis. **A** and **b**—proton density (PD)-weighted turbo spin echo (TSE) Spectral Attenuated Inversion Recovery (SPAIR), **c** and **d**—T2-weighted TSE SPAIR

The ACJ cavity was visible simultaneously in the three anatomical planes in 47.5% of the re-reviewed examinations (Table 3). If the ACJ was visible in two planes, it was most often the axial and coronal planes (21.1%) (Table 3). ACJ was visible only in only one plane in 11.8%, most often the axial plane (6.9%) (Table 3). In total, the ACJ cavity was visible 84.8% of the time in the axial plane, 77.9% in the coronal plane and 71.1% in the sagittal plane (Table 3), and the differences in visibility were statistically significant. In *n* = 6 (2.9%) of the examinations, the joint cavity was not visible in any of the three planes (Table 3). Examples of visible and non-visible joint cavities are shown in Figs. 2, 3, 4, 5.

**Table 3:** Number and percent of examinations where the acromioclavicular joint cavity was visible in all planes, two planes and one plane

Visibility vs. plane or planes	Visibility, *n*	Visibility, %	95% CI	
			Upper	Lower
Simultaneously in the axial, coronal and sagittal plane	97	47.5%	41.6%	54.5%
Axial plane and coronal plane	43	21.1%	8.9%	33.3%
Axial plane and sagittal plane	24	11.8%	– 1.2%	24.7%
Coronal plane and sagittal plane	10	4.9%	-8.5%	18.3%
Only axial plane	14	6.9%	– 6.9%	19.7%
Only coronal plane	6	2.9%	– 10.6%	16.5%
Only sagittal plane	4	2.0%	– 11.7%	15.6%
Not visible in any plane	6	2.9%	– 10.6%	16.5%
*In total*				
Axial planes	173	84.8%	89.9%	80.1%
Coronal planes	159	77.9%	84.4%	72.3%
Sagittal planes	145	71.1%	77.2%	65.5%

*CI *confidence interval

The mean angle was 41.1 deg. between the ACJ and the glenoid cavity in the axial plane, 34.4 deg. in the coronal plane and 52.5 deg. in the sagittal plane, and values were in the CI 95% (Table 4). The agreement between observers was measured using ICC and interpreted according to Landis and Koch [13]. Fair agreement was assessed in angle measurements in the sagittal plane (ICC 0.38, 95% CI (0.11–0.68)), moderate in the coronal plane (ICC 0.61, 95% CI (0.27–0.82)) and substantial in the axial plane (ICC 0.72, 95% CI (0.28–0.89)). SEM was lowest in the axial plane and highest in the sagittal plane (Table 4). The distribution of acromion types and the degree of agreement between observers are presented in Table 5. Moderate agreement was assessed for types 1 and 2 and fair for type 4. The most common acromion was type 2 (Table 5).

**Table 4:** Angles between the acromioclavicular joint and the glenoid cavity and intraclass correlation coefficient

Angle	Degrees
Mean axial angle (°)	41.1 CI 95% = 39.7, 42.5
ICC	0.72
(95% CI)	(0.28–0.89)
SEM	2.1
Mean coronal angle (°)	33.4 CI 95% = 31.3, 35.5
ICC	0.61
(95% CI)	(0.27–0.82)
SEM	2.3
Mean sagittal angle (°)	52. 5 CI 95% = 50.1, 54.9
ICC	0.38
(95% CI)	(0.11–0.68)
SEM	2.4

*CI *confidence interval, *ICC *intraclass correlation coefficient, *SEM *standard error measurement

**Table 5:** Types of the acromion and agreement between observers

Type of acromion	*n* (%)	% of agreement	Cohen's kappa (κ)
Type 1	15 (7.4)	75.49	0.41
Type 2	176 (86.3)	82.8	0.49
Type 3	0 (0)	×	×
Type 4	13 (6.4)	81.8	0.31

Acromion type 2 was most common and seen in 86.3% of the examined shoulders. Acromion type 1 was seen in 7.35% of the examinations, while type 4 was seen in 6.4% of the shoulders. Acromion type 3 was not noticed in any of the examined shoulders (Table 5). No testing was done regarding acromion types, as there were not enough samples that were not acromion type 2. Fair to moderate agreement according to Landis and Koch [13] was noticed in the acromion type evaluation (Table 5). The highest agreement was noticed in type 2 and the lowest in type 4 (Table 5). In four cases, *n* = 4 (2%) os acromiale was identified (male *n* = 3 [*n* = 2 left side, *n* = 1 right side], female *n* = 1 left side).

When comparing the group of patients with a visible joint cavity (group 1) with the group of patients where the joint cavity is not visible in the three anatomical planes (group 2), there was no significant difference in gender distribution or mean age (Table 6).

**Table 6:** Comparison of mean age and gender distribution between group 1 (visible joint cavity in three anatomical planes) and group 2 (non-visible joint cavity in three anatomical planes)

	Group 1	Group 2	*i*
Mean age	24.12	24.22	0.8413
Males	74	71	0.1253
Females	23	36	0.1253

**Fig. 6:**
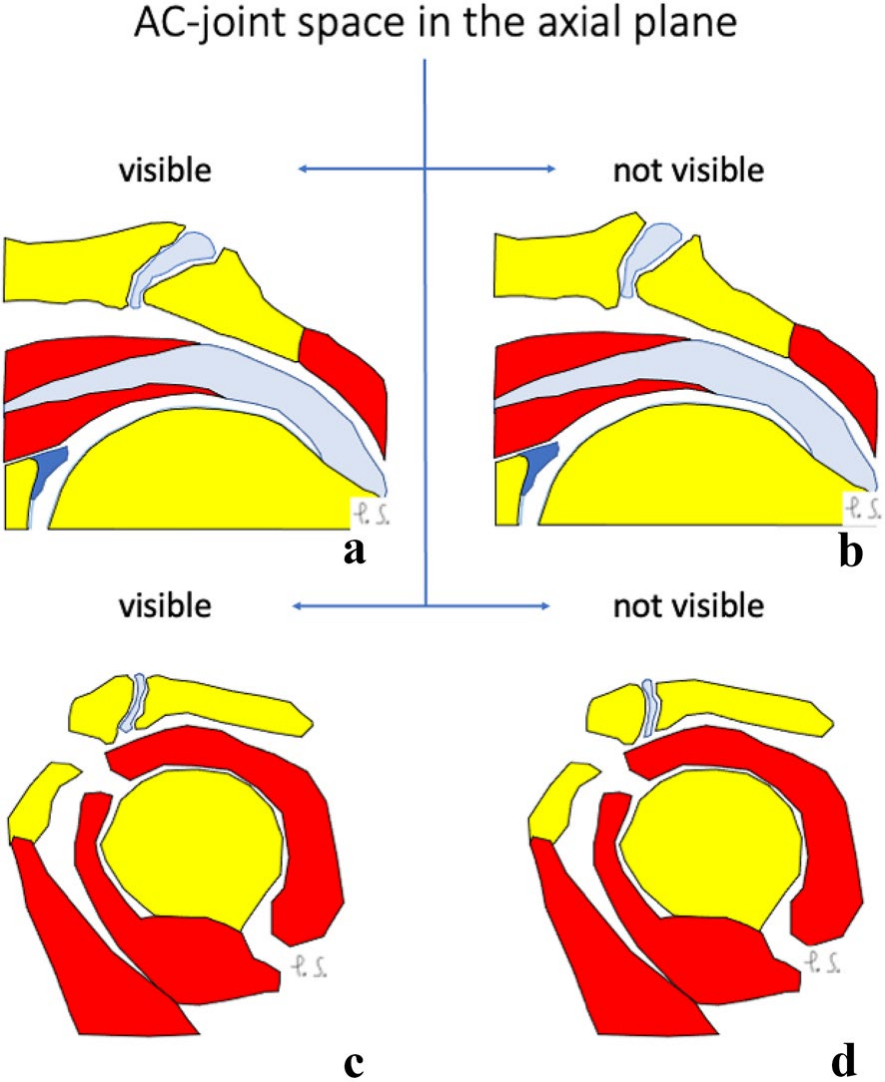
Configuration of the acromioclavicular joint space on the coronal (**a** and **b**) and sagittal planes (**c** and **d**) when the joint cavity is visible (**a** and **c**) or not (**b** and **d**) in the axial plane. Larger values of the acromioclavicular joint space angle in the coronal (**b**) and sagittal (**d**) planes were noticed when the joint cavity was not visible in the axial plane. Differences in values of angles of the acromioclavicular joint were statistically significant (Table 7). Figure prepared by PS

When comparing the group where the joint cavity is visible in the axial plane and the group where it is not, there were statistically significant differences between the mean angles in both the coronal and sagittal planes (Table 7). In patients where the joint cavity was visible in the axial plane, both the coronal and sagittal angles between the ACJ and the glenoid tend to be greater comparing to group where the joint cavity was not visible, differences were statistically significant (Table 7 and Fig. 6).

**Table 7:** Mean angles in different sections when the joint cavity is visible or not visible in axial view

Joint cavity in the axial plane	The angle in the axial plane (degrees)	The angle in the coronal plane (degrees)	The angle in the sagittal plane (degrees)
Visible	41.12	41.39	58.83
Not visible	Not analyzed	32.10	51.41
*p*	Not analyzed	< 0.01	< 0.001

**Fig. 7:**
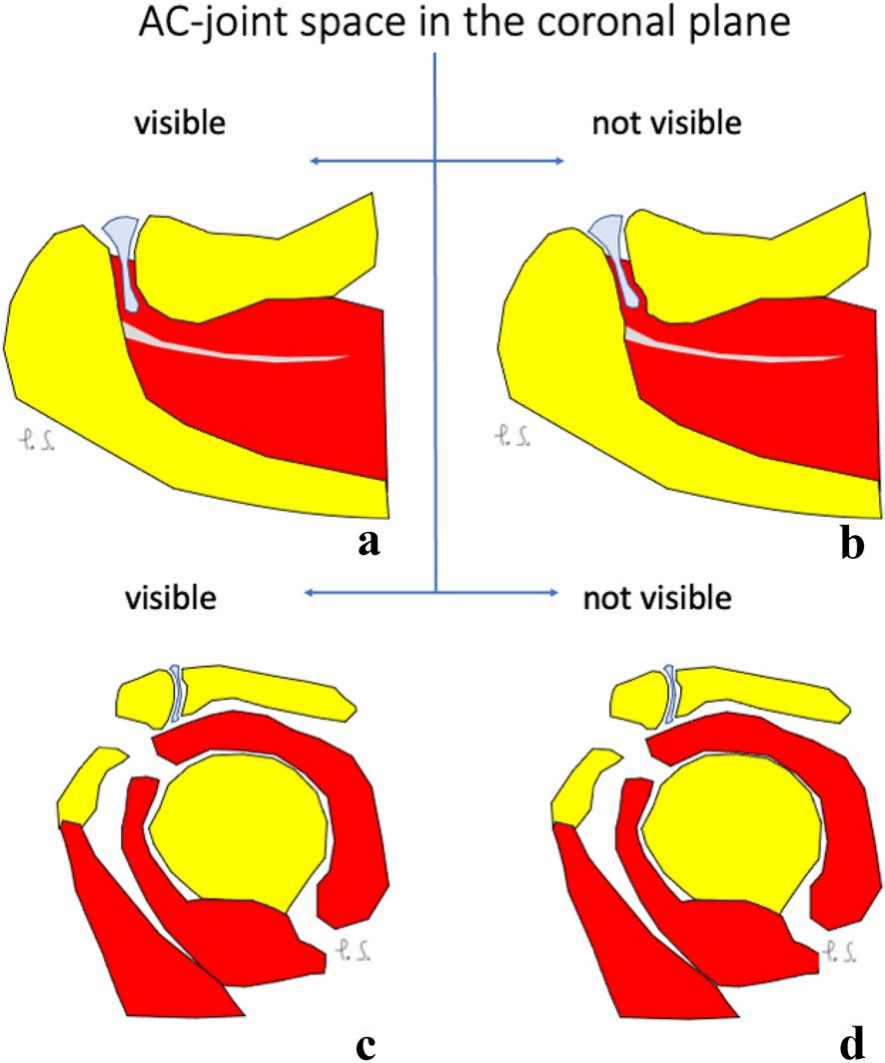
Configuration of the acromioclavicular joint space on the axial (**a** and **b**) and sagittal planes (**c** and **d**) when the joint cavity is visible (**a** and **c**) or not (**b** and **d**) in the coronal plane. Larger values of the acromioclavicular joint space angle in the axial (**b**) plane are noticed when the joint cavity is not visible in the coronal plane. Differences in values of angles of the acromioclavicular joint were not statistically significant (Table 8). Figure prepared by PS

In patients where the joint cavity was visible in the coronal plane, the angle between the ACJ and the glenoid measured on the axial plane tends to be greater compared to the group where the joint cavity was not identifiable; however, it was not statistically significant (Table 8 and Fig. 7). The mean angle value in the sagittal plane was almost the same (Table 8 and Fig. 7).

**Table 8:** Mean angles in different sections when the joint cavity is visible or not visible in the coronal view

The joint cavity in the coronal plane	The angle in the axial plane (degrees)	The angle in the coronal plane (degrees)	The angle in the sagittal plane (degrees)
Visible	40.55	33.39	52.48
Not visible	43.31	Not analyzed	52.52
*p*	0.12	Not analyzed	0.98

**Fig. 8:**
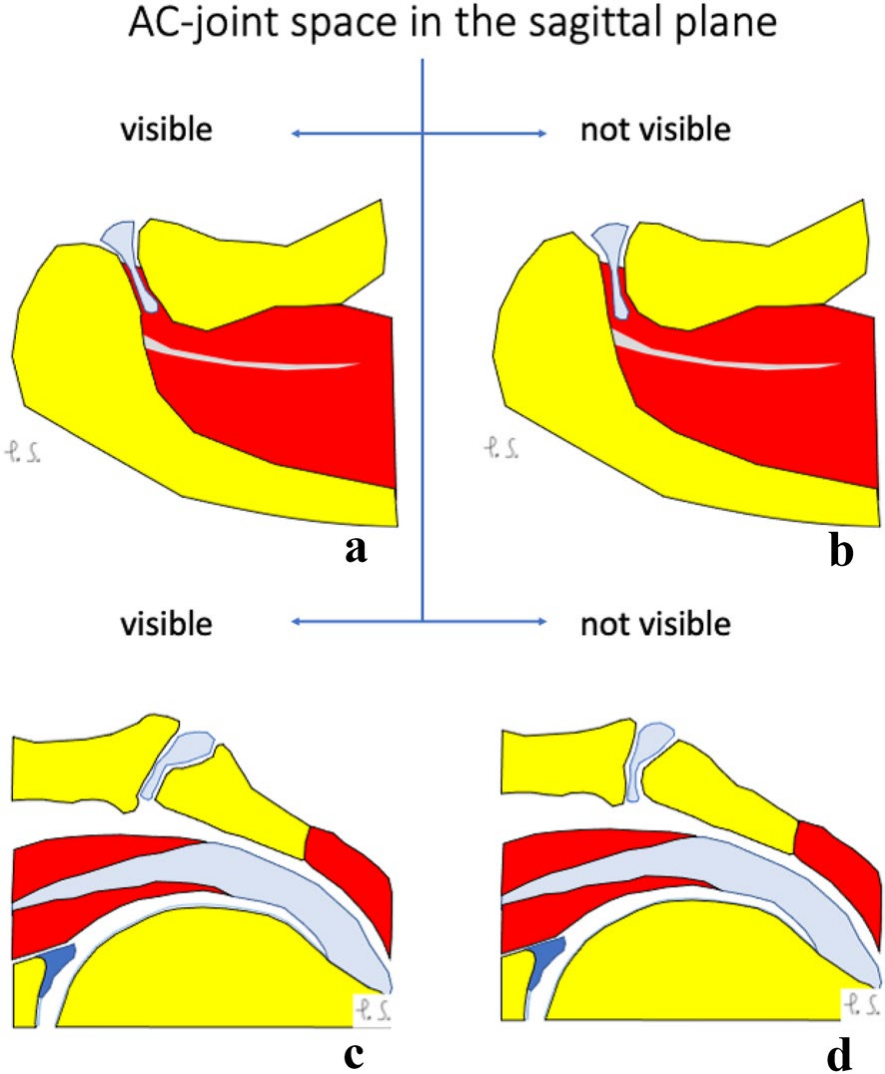
Configuration of the acromioclavicular joint space on the axial (**a** and **b**) and coronal planes (**c** and **d**) when the joint cavity was visible (**a** and **c**) or not (**b** and **d**) in the sagittal plane. Larger values of the acromioclavicular joint space angle in the axial plane (**a**) were noticed when the joint cavity was visible in the sagittal plane. Larger values of the acromioclavicular joint space angle in the coronal plane (**d**) were noticed when the joint cavity was not visible in the sagittal plane. Differences in values of angles of the acromioclavicular joint were statistically significant (Table 9). Figure prepared by PS

In patients where the joint cavity could be seen in the sagittal plane, the angles between the ACJ and the glenoid measured in the axial and coronal planes tend to be greater compared to patients where the joint cavity was not visible in the sagittal plane, differences were statistically significant (Table 9 and Fig. 8).

**Table 9:** Mean angles in different sections when the joint cavity is visible or not visible in sagittal view

The joint cavity in the sagittal plane	The angle in the axial plane (degrees)	The angle in the coronal plane (degrees)	The angle in the sagittal plane (degrees)
Visible	42.27	34.89	52.49
Not visible	38.23	29.45	Not analyzed
*p*	0.01	0.02	Not analyzed

**Fig. 9:**
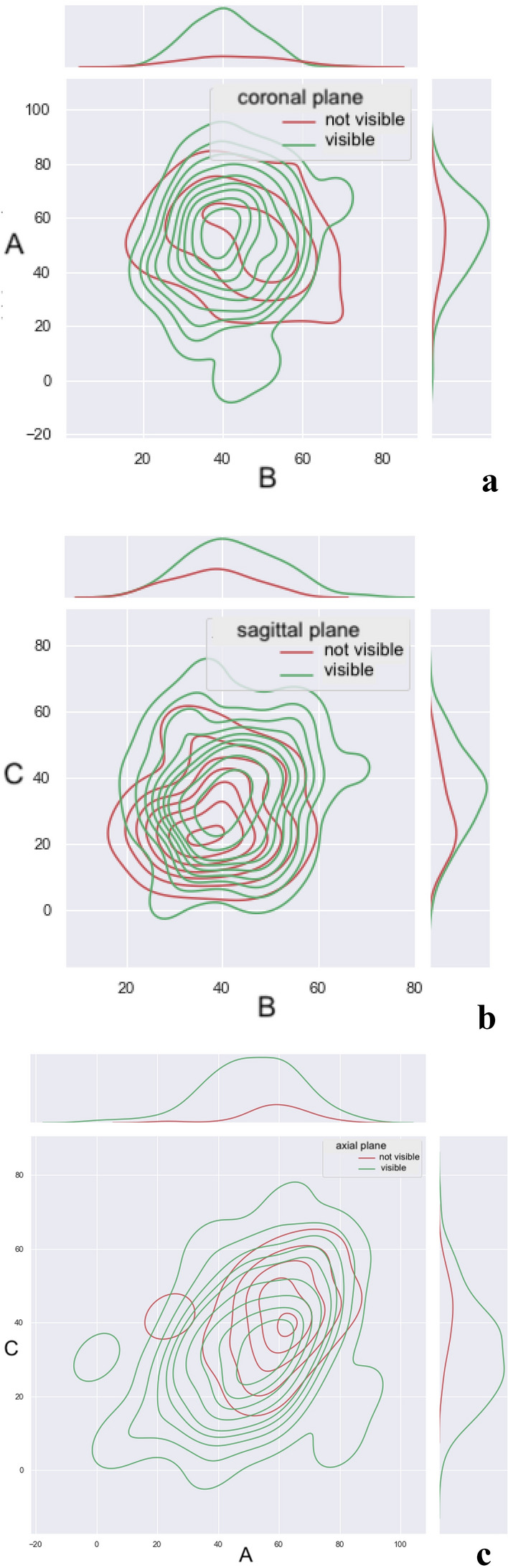
The plots show the difference in the angles of the shoulder–clavicular joint with respect to the glenoid cavity in the sagittal (**A**), transverse (**B**) and frontal (**C**) planes in groups where the shoulder–clavicular joint is visible or not in the coronal plane (**a**), the sagittal plane (**b**) and the axial plane (**c**). The figure was done in SPSS Statistics software version 22.0

As can be seen from the above, the configuration of the ACJ differs between groups where the joint cavity is visible or not (Figs. 6, 7, 8 and 9).

There was no statistically significant difference in mean angles between the ACJ and the glenoid when comparing males and females (Table 10).

**Table 10:** Comparison of the angles between the acromioclavicular joint and the glenoid in females and males

	Females	Males	*p*
The angle in the axial plane (degrees)	40.56	41.35	0.95
The angle in the coronal plane (degrees)	33.20	33.46	0.79
The angle in the sagittal plane (degrees)	49.33	53.57	0.16

## Discussion

The most important finding of this study was that in most of the scans it was not possible to assess the joint space of ACJ in three anatomical planes because of the anatomical variations. The other finding was that variability of the ACJ influences its visibility on the MRI of the shoulder. To the best of the author's knowledge, no study has been conducted to investigate the influence of ACJ anatomical variations on its visibility on MRI.

To the best of our knowledge, no one before has used the method of calculating the angle between ACJ and the glenoid cavity. We believe that this angle, measured in different planes, can be a good indicator of the anatomical variability of the ACJ. The relatively good agreement between the results of different observers is promising.

MRI of the shoulder has a well-established position in the evaluation of patients with ACJ pain [1]. When no pathology is shown in the ACJ, this does not leave out that a patient's pain originates there because visualization in only one or two planes does not allow for a complete evaluation. Appropriate visualization of the ACJ is the basis for the ability to its radiological evaluation, which can be challenging with X-rays [1]. According to the previous studies, MRI results may change the Rockwood type assessed with X-ray [20]. Due to its high spatial resolution, MRI is an excellent method to assess tissue contrast, and thus, it is the most commonly used imaging method to evaluate all components of a shoulder joint [11]. However, the difficulty of evaluating the ACJ with a standard shoulder MRI scan has been recognized by other authors [5, 23] and is now also confirmed in the current study.

The visibility of the joint space of ACJ was not dependent on gender or age which means that the problem with the assessment of ACJ occurs regardless of gender. This was expected, as there is no reason why males and females would differ at this point in this relatively young group. It is also not surprising that there is no difference within a group with such similar ages. However, if one would evaluate the visibility of the ACJ of our group of patients 18–31 years old with a group of older patients, such as those aged between 51 and 65 years, a difference could be expected. In older patients, an anatomical difference can be expected due to more significant degeneration [17].

We showed that the angles between the ACJ and the glenoid differed significantly between patients where the joint cavity was visible in the axial view and those where it was not visible. The same applies to the sagittal view. Significant anatomical variability in the position and orientation of the joint cavity of the ACJ may have a developmental origin. In the ACJ, two bones with two different ossification patterns articulate. The clavicle has membranous ossification, while the scapula has cartilaginous ossification. The acromion and the clavicula begin to develop quite early at Carnegie stage 19–20, which corresponds to the blastemal and chronogeneous period [24]. This observation indicates that the ACJ is important from a developmental and evolutionary point of view. The location of the ACJ at the alternating extremities of two different bones may predispose one to high anatomical variability in the developmental background [24].

Degenerative changes may also change bone morphology, especially in those parts that form joints, but in the examined group, degenerative changes usually do not occur. On the other hand, overload changes may occur in the examined group, and they usually do not cause changes in the structure of the bones, but rather bone marrow edema [26]. The anatomical variants of the ACJ may be an important factor that influences how much the ACJ is visible.

No testing was done concerning how the type of acromion or os acromiale and ACJ visibility were correlated. This was because of the small number of examinations showing other acromion types than type II and low number of os acromiale. To do that type of testing, a larger sample of patients would be needed. This could be of interest, since our results show that anatomical attributes influence the visibility of the joint cavity. In the present study, the Bigliani classification of the acromion was used [16]. The reason is the widespread use of this classification, communication among clinicians and the known relationship between acromial morphology and rotator cuff tendinopathy. The agreement between observers of the individual types of the acromion has been repeatedly studied, with quite mixed results. Cohen's kappa ranged from slight to excellent agreement in previous studies [16].

In our sample, we did not find any acromion type III. This was unexpected, since the frequency observed by other authors is usually around 10% [8]. However, as mentioned earlier, the interobserver reliability of this classification is poor [16]. Some of those whom we classified as acromion type II might have been classified as acromion type III by another observer. In an article published in 2003 [4], there is an example of acromion type III (Fig. eleven in the referred article) that we would have classified as acromion type II. However, the results of previous studies are often contradictory. Results obtained in the current study may be related to the methodology. In our study, we only assessed MRI. It is difficult to unequivocally say whether the application of radiography would significantly change the results. According to previous studies, radiography had a fair agreement in ACJ evaluation and was superior to any single MRI image. However, a combination of two MRI images showed better agreement than radiography [15].

Os acromiale can cause the development of subacromial impingement because of the mobile connection with the acromion. Previous studies have not analyzed whether the acromioclavicular joint configuration and the presence of the os acromiale are somehow related to each other. In our cohort frequency of the os acromiale was lower than the previously reported average, but it was in the lower frequency range [6, 27].

Anatomical ACJ variants are difficult to study due to a few fixed points. In our study, we chose the glenoid cavity because of its fixed location. Unfortunately, there is relatively little research regarding the AC; hence, it is difficult to clearly discuss the angles.

The most important finding in our study is the fact that fewer than half of the MRI scans visualize the ACJ in three planes. This implicates that the standard MRI shoulder protocol is not fully sufficient to examine the ACJ. We have shown that the anatomical variants of the ACJ, measured as the angle between the joint cavity and the glenoid, differs between patients where the joint cavity is visible and patients where it is not visible. Consequently, we can see the need for a new protocol for examining the ACJ that is based on the patient's anatomy. The current MRI protocol for examining the shoulder, which is created to visualize the rotator cuff, is also based on the rotator cuff anatomy in the examined patient, since the coronal cut is put along with the supraspinatus muscle. In analogy to this, we could see a need for a protocol for examining the ACJ where the sections are dependent on the ACJ in the patient. The use of 3D sequences with isovolumetric voxels can reduce the impact of various configurations of the ACJ cavity. 3D sequences are often used in the ankle joint to evaluate ligaments and cartilage. The evaluations were significantly better on 3D sequences compared to 2D [21] or on the stress MRI [10]. Anatomical variations are common in both the ACJ and the ankle joint. Since there are no differences in joint cavity visibility between males and females, nor between patients of different ages within our group, it should be sufficient to have one new protocol without adjustments dependent on the patients' age or gender.

The limitations of the current study should be acknowledged. To improve measurement accuracy, a special protocol with thin layers or with 3D sequences can be used. Measurement errors may be related to slide thickness and the angle of the section. The retrospective character and the lack of surgical correlation may also be limitations of this study. Another limitation is the lack of information about the subjects' height, weight and body mass index.

## Conclusion

The standard routine MRI protocol for examining the shoulder region could not visualize the ACJ in three anatomical planes in more than half of the re-reviewed examinations of patients aged 18–31 years. This should be known for any doctor referring patients for MRI scans of the shoulder, since an examination without any pathological findings in the ACJ does not axiomatically leave out that the source of the patient's pain is located there.

Whether the joint was visible was not dependent on the gender or age of the patient. However, we could see that the anatomy of the ACJ measured as the angle between the joint cavity and the glenoid played a role in deciding whether the ACJ was visible. The implication is consequently that all patients aged 18–31 years could benefit from a new adjusted protocol for examining the ACJ. The standard protocol used for MRI of the shoulder joint is not sufficient for full assessment of the ACJ in the 18–31-year-old group.

## Data Availability

The datasets generated during and/or analyzed during the current study are available from the corresponding author on reasonable request.

## Abbreviations

ACJ: Acromioclavicular joint
CI: Confidence interval
FOV: Field of view
ICC: Intraclass correlation coefficient
MRI: Magnetic resonance imaging
PACS: Picture archiving and communication system
PD: Proton density
SEM: Standard error measurement
SPAIR: Spectral adiabatic inversion recovery
TE: Echo time
TR: Repetition time
TSE: Turbo spin echo

## References

[1] Alyas F, Curtis M, Speed C (2008). MR imaging appearances of acromioclavicular joint dislocation. Radiographics.

[2] Choo HJ, Lee SJ, Kim JH (2013). Can symptomatic acromioclavicular joints be differentiated from asymptomatic acromioclavicular joints on 3-T MR imaging?. Eur J Radiol.

[3] Depalma AF (1963). Surgical anatomy of acromioclavicular and sternoclavicular joints. Surg Clin North Am.

[4] Ernberg LA, Potter HG (2003). Radiographic evaluation of the acromioclavicular and sternoclavicular joints. Clin Sports Med.

[5] Fialka C, Krestan CR, Stampfl P (2005). Visualization of intraarticular structures of the acromioclavicular joint in an ex vivo model using a dedicated MRI protocol. AJR Am J Roentgenol.

[6] Fischer CS, Floss M, Ittermann T (2022). Os acromiale: prevalence and associated patient-related factors-a population-based study of three thousand and fifty participants. Int Orthop.

[7] Flores DV, Goes PK, Gómez CM (2020). Imaging of the Acromioclavicular joint: anatomy, function, pathologic features, and treatment. Radiographics.

[8] Getz JD, Recht MP, Piraino DW (1996). Acromial morphology: relation to sex, age, symmetry, and subacromial enthesophytes. Radiology.

[9] Heers G, Götz J, Schubert T (2007). MR imaging of the intraarticular disk of the acromioclavicular joint: a comparison with anatomical, histological and in-vivo findings. Skeletal Radiol.

[10] Izadpanah K, Winterer J, Vicari M (2013). A stress MRI of the shoulder for evaluation of ligamentous stabilizers in acute and chronic acromioclavicular joint instabilities. J Magn Reson Imaging.

[11] Kijowski R, Gold GE (2011). Routine 3D magnetic resonance imaging of joints. J Magn Reson Imaging.

[12] Koo TK, Li MY (2016). A guideline of selecting and reporting intraclass correlation coefficients for reliability research. J Chiropr Med.

[13] Landis JR, Koch GG (1977). The measurement of observer agreement for categorical data. Biometrics.

[14] Ludewig PM, Phadke V, Braman JP (2009). Motion of the shoulder complex during multiplanar humeral elevation. J Bone Joint Surg Am.

[15] Mayerhoefer ME, Breitenseher MJ, Roposch A (2005). Comparison of MRI and conventional radiography for assessment of acromial shape. AJR Am J Roentgenol.

[16] McLean A, Taylor F (2019). Classifications in brief: bigliani classification of acromial morphology. Clin Orthop Relat Res.

[17] McLean M, Hoban K, Gupta R (2019). The epidemiology of acromioclavicular joint excision. J Orthop Surg (Hong Kong).

[18] Menge TJ, Boykin RE, Bushnell BD (2014). Acromioclavicular osteoarthritis: a common cause of shoulder pain. South Med J.

[19] Minagawa H, Yamamoto N, Abe H (2013). Prevalence of symptomatic and asymptomatic rotator cuff tears in the general population: From mass-screening in one village. J Orthop.

[20] Nemec U, Oberleitner G, Nemec SF (2011). MRI versus radiography of acromioclavicular joint dislocation. AJR Am J Roentgenol.

[21] Notohamiprodjo M, Kuschel B, Horng A (2012). 3D-MRI of the ankle with optimized 3D-SPACE. Invest Radiol.

[22] Petersson CJ, Redlund-Johnell I (1983). Radiographic joint space in normal acromioclavicular joints. Acta Orthop.

[23] Schaefer FK, Schaefer PJ, Brossmann J (2006). Experimental and clinical evaluation of acromioclavicular joint structures with new scan orientations in MRI. Eur Radiol.

[24] Tanaka S, Sakamoto R, Kanahashi T (2020). Shoulder girdle formation and positioning during embryonic and early fetal human development. PLoS ONE.

[25] van der Windt DA, Koes BW, de Jong BA (1995). Shoulder disorders in general practice: incidence, patient characteristics, and management. Ann Rheum Dis.

[26] Veen EJD, Donders CM, Westerbeek RE (2018). Predictive findings on magnetic resonance imaging in patients with symptomatic acromioclavicular osteoarthritis. J Shoulder Elbow Surg.

[27] You T, Frostick S, Zhang WT (2019). Os Acromiale: Reviews and Current Perspectives. Orthop Surg.

